# Influence of occupational exposure on hyperuricemia in steelworkers: a nested case–control study

**DOI:** 10.1186/s12889-022-13935-x

**Published:** 2022-08-08

**Authors:** Yuanyu Chen, Yongzhong Yang, Ziwei Zheng, Hui Wang, Xuelin Wang, Zhikang Si, Rui Meng, Guoli Wang, Jianhui Wu

**Affiliations:** 1 School of Public Health, Caofeidian New Town, North China University of Science and Technology, No.21 Bohai Avenue, Tangshan City, Hebei Province 063210 People’s Republic of China; 2 Hebei Province Key Laboratory of Occupational Health and Safety for Coal Industry, North China University of Science and Technology, Tangshan, Hebei People’s Republic of China

**Keywords:** Hyperuricemia, Occupational hazards, Steelworkers, Nested case–control study

## Abstract

**Background:**

Occupational exposure may be associated with an increased risk of developing hyperuricemia. This study sheds lights on the association between occupational exposure and hyperuricemia in steelworkers.

**Method:**

A nested case–control study was conducted within a cohort of workers in steel companies to explore the association between occupational exposure and hyperuricemia. The case group consisted of a total of 641 cases of hyperuricemia identified during the study period, while 641 non-hyperuricemia subjects with the same age and gender distribution were randomly selected from the cohort as the control group.

**Results:**

The incidence rate of hyperuricemia among workers in the steel company was 17.30%, with an incidence density of 81.32/1,000 person-years. In comparison to the reference group, the risks of developing hyperuricemia for steelworkers undergoing ever shifts, current shifts, heat exposure, and dust exposure were 2.18 times, 1.81 times, 1.58 times and 1.34 times higher respectively. The odds ratios (ORs) and 95% confidence intervals (*CIs*) were 1.87(1.12–3.13) and 2.02(1.21–3.37) for the cumulative number of days of night work at 0–1,972.80 and ≥ 1,972.80 (days), respectively. Compared to the group with the cumulative heat exposure of 0 (°C/year), the ORs (95% *CI*) for the risk of developing hyperuricemia in the groups with the cumulative heat exposure of 0–567.83 and ≥ 567.83 (°C/year) were 1.50(1.02–2.22) and 1.64(1.11–2.43), respectively. The OR (95% *CI*) for the risk of developing hyperuricemia was 1.56(1.05–2.32) at the cumulative dust exposure of ≥ 30.02 (mg/m^3^/year) compared to that at the cumulative dust exposure of 0 (mg/m^3^/year). Furthermore, there was a multiplicative interaction between heat exposure and dust exposure in the development of hyperuricemia.

**Conclusion:**

Shift work, heat, and dust are independent risk factors for the development of hyperuricemia in steelworkers. Additionally, there is a multiplicative interaction between heat exposure and dust exposure in the development of hyperuricemia. Interventions for shift work, heat and dust may help to reduce the incidence rate of hyperuricemia and improve the health of steelworkers.

## Background

Hyperuricemia (HUA) refers to a group of metabolic disorders. To be more specific, the concentration of uric acid in the blood is too high due to chronic impairment of purine nucleotide metabolism and, or abnormal excretion of uric acid in the body [1]. In the early stages, hyperuricemia is characterized only by elevated blood uric acid concentration and is highly insidious. Gouty arthritis and, in severe instances, renal impairment can develop when the blood is saturated with urate [2]. Studies have confirmed that hyperuricemia not only causes gouty arthritis and renal impairment but is also associated with type 2 diabetes, hypertension, coronary artery disease, endothelial dysfunction, and metabolic syndrome [3–7]. A study of 36,348 adults showed that the prevalence of hyperuricemia among Chinese adults was 8.4% in 2009–2010 [8]. In 2015, a mate-analysis conducted by Liu R et al. involving 44 epidemiological surveys in 16 provinces, municipalities, and autonomous regions in the mainland of China, revealed that the overall prevalence of hyperuricemia in China was 13.3% [9]. In addition, the prevalence of hyperuricemia among US adults, significantly higher than the results of the 1988–1994 survey (18.2%), rises to 20.1% according to the 2007–2016 US National Health and Nutrition Examination Survey [10]. Thus, it appears that the prevalence of hyperuricemia is increasing every year.

Studies have shown that heat exposure can lead to kidney damage [11], which in turn can affect the metabolism of uric acid. Roncal-Jimenez CA et al. carried out an animal experiment in which mice exposed to heat for 5 weeks had higher serum uric acid levels, indicating that heat exposure is associated with elevated uric acid in the serum [12]. A large case–control study was conducted, and the results revealed a link between inorganic dust exposure and gout [13]. In addition, a Russian study showed a higher prevalence of hyperuricemia in oil press workers exposed to chronic noise than that in the general population [14]. Different production processes in steel plants involve a variety of occupational hazards such as heat, noise, dust, and long irregular shifts, all of which can be hazardous to the health of workers in steel companies and give rise to various diseases. LI X et al. conducted a survey of steelworkers and made an analysis. They found that the prevalence of hyperuricemia among steelworkers was 35.9% [15], which was higher than that of the general population. The factors that affect hyperuricemia have been extensively studied [16–19]. However, these studies have mainly focused on social behavior, dietary habits, and genetic factors. There have been very few investigations into the influence of occupational hazardous variables on hyperuricemia. Hence, the current study was carried out to look into the connection between occupational exposure and the development of hyperuricemia in workers at a steel company. Besides, the interactions between occupational hazards were examined.

## Methods

### Population study

The participants in this study were workers of a steel company who underwent occupational health screening at Hongci Hospital, Tangshan City, Hebei Province, China, from March 2017 to September 2017. Cohort inclusion criteria: formally employed workers, at least one year of service. Cohort exclusion criteria: hyperuricemia and refusal to participate in this cohort study. A total of 4,247 workers from the steel company were included in the cohort. A follow-up survey of a total of 3,706 steelworkers between March 2019 and September 2019 was carried out, with a 12.74% missing rate. Workers in the cohort with new-onset hyperuricemia in steel companies are regarded as the case group. From this cohort population, each new-onset patient was matched with a worker in the steel company without hyperuricemia as a control according to the principle of matched design (the same sex and age) to form a control group. A total of 641 case and control pairs were eventually included as samples after those with incomplete information were excluded.

All participants have read and signed the informed consent form. The subject was approved by the Medical Ethics Committee of the North China University of Technology (No.15006).

### Questionnaire

A one-on-one survey was conducted by uniformly trained enumerators with a questionnaire designed by the subject group. The contents of the questionnaire included sex, age, household size, monthly household income, education level, marital status, smoking status, drinking status, tea status, physical exercise, type of work, change in type of work, shift work, type of shift, change in shift work, change in working hours, daily working hours, monthly rest time, and personal disease history. The participants were also asked about the frequency of food intake (0 day per week, 1–2 days per week, 3–6 days per week, and 7 days per week). Food was divided into different groups: vegetables, fruits, meat, eggs, dairy products, soy products, and seafood.

### Physical examination

The participants took off their shoes when their height and weight were measured through the ultrasound height and weight measuring device. Measurements were taken three times and averaged. The body mass index (BMI) was calculated based on the measurement. The participant was instructed not to drink tea, coffee, alcohol or other beverages that may affect the results of blood pressure. The participant was asked to have a five-minute break before the blood pressure measurement and to take the measurement three times with an interval of not less than three minutes.

### Laboratory examination

Subjects were required to fast for 12 h. Fasting blood and morning urine were collected by the Laboratory Department of Tangshan Hongci Hospital before 9 a.m. the next day. Blood, urine, and blood biochemistry were examined by specialist physicians.

### On-site hygienic investigation

The on-site hygienic survey involved heat, dust, and noise.

The measurement tool for temperature is Wet Bulb Black Globe Temperature Gauge (WBGT). According to relevant standard [20], the temperature should be measured during the hottest season of the year. Temperatures were measured at different workplaces in consideration of the specific conditions of the steel production unit. Three to six measurement points were selected for each workplace, and the test was repeated three times at each measurement point, with the average taken as the final result.

Dust was measured with a dust sampler. The sampling points were chosen according to relevant standard [21] and specific conditions of the workshop. The sample collection time for each sampling point was 45 min, and the flow rate of the dust sampler was set at 40 L/min. The calculation formula is as follows:

1 $$\begin{array}{c}C=\frac{\mathrm{m}2-\mathrm{m}1}{\mathrm{Q}\times \mathrm{t}}\times 1000\#\end{array}$$

where: C- dust concentration, mg/m.^3^

m2—the mass of the filter membrane after sampling, mg.

m1—the mass of the filter membrane before sampling, mg.

Q—flow rate, L/min.

t—sampling time, min.

Noise testing was carried out according to relevant standards [22, 23] and specific circumstances of the workplace. When the noise distribution in the workshop was uneven, the noise was divided into different sound zones according to the sound level and two test points were set up in each zone. When the noise distribution in the workshop was relatively even (the sound level difference is less than or equal to 3 dB (A)), three measurement points were set up. The average value was taken as the final result after measurement. The calculation formula for sound level measurement is as follows:

2 $${L}_{Aeq,T}=10\text{lg(}\frac{1}{T}{\sum }_{i=1}^{n}{T}_{i}{10}^{0.1{L}_{Aeq,{T}_{i}}})$$

where: ${L}_{Aeq, {T}_{i}}$- equivalent sound level during the time period T_i_.

${L}_{Aeq, T}$—equivalent sound level for a full day.

n—the total number of periods.

T—the duration of each period.

T_i_- i period of time.

Cumulative exposure measurement (CEM) for steelworkers is calculated based on the results of the on-site hygienic survey, combined with the change in work status and duration of occupational exposure. The formula is as follows:

3 $$\mathrm{CEM}={\mathrm{L}}_{1} {\mathrm{T}}_{1}+ {\mathrm{L}}_{2} {\mathrm{T}}_{2}\dots \dots +{\mathrm{L}}_{\mathrm{n}} {\mathrm{T}}_{\mathrm{n}}$$

where: L_n_ is the average exposure to the target harmful factor over a period of time T_n_.

### Definition and grouping of indicators

Those having a blood uric acid value greater than or equal to 7.0 mg/dL in men and 6.0 mg/dL in women, as well as previous or ongoing gout treatment, were diagnosed with hyperuricemia [24]. Never smokers were defined as those who had never smoked from birth to the time of the survey. Former smokers were defined as those who had previously smoked but had quit smoking for 6 months or longer as of this survey. People who had smoked at least 1 cigarette per day for six months or longer as of the survey were defined as current smokers. People who drank alcohol more than twice a week, regardless of the type of alcohol, and who had done so for more than a year were considered to be drinking. The frequency of food consumption was divided into four categories: never (0 day per week), occasionally (1–2 days per week), frequently (3–6 days per week) and daily (7 days per week). In this study, the International Physical Activity Questionnaire (IPAQ) was used to analyze the physical activities of steelworkers [25]. Physical activities were classified into light, moderate, and heavy activities based on intensity, frequency, and overall weekly physical activity level. Shift work is a system of irregular working hours in which one or more teams perform tasks continuously for 24 h by working in shifts without stopping [26]. The cumulative number of days of night work represents the total number of days of night work done by workers in the steel plant as of the date of the survey. According to relevant standard [20], work with a productive heat source and WBGT ≥ 25 °C was defined as heat-exposed work. Dust exposure was defined based on the type of work, the work environment, and the findings of the site hygiene [21]. Exposure to noise was defined as workers who were exposed to a noisy environment where the 8 h/d or 40 h/week equivalent A-weighted sound pressure level is ≥ 80 dB, which may be harmful to health and hearing [27].

### Statistical methods

Continuous variables were described by means and standard deviations, and the differences between groups were obtained through Student’s t-test. The categorical variables were expressed through the number of individuals (%), and χ^2^ tests were used for comparisons between groups. Multifactorial analyses were performed and multiplicative interactions between occupational hazard factors were explored with the help of conditional logistic regression models. Additive interactions were assessed using the attributable proportion of interaction (AP), the relative excess risk of interaction (RERI), and the synergy index (SI), calculated using the Excel spreadsheet by Andersson et al. [28]. The AP is the proportion of the risk due to the interaction in the doubly exposed group. When RERI is positive, it indicates increased risk due to the additive interaction. SI can be interpreted as the ratio of an increased risk due to both exposures to the sum of individual increased risks.

All statistical analyses were performed by dint of IBM SPSS 24.0 and Excel 2019. *P* < 0.05 was regarded as significant for two-sided tests.

## Results

### Basic information

A total of 3,706 workers (3,352 males and 354 females) were followed up in the steel enterprise. The follow-up period was (25.88 ± 1.68) months. 641 new cases of hyperuricemia (587 males and 54 females) were found among the 3,706 workers in the steel company, with a mean age of (44.67 ± 7.58) years. The case and control groups have the same sex and age composition because the same sex and age were required and 1:1 matching was used according to the matching principle. The two groups were comparable with the same basic data. The incidence rate was 17.30% (17.51% for males and 15.25% for females). The incidence density was 81.32/1,000 person-years (82.35/1,000 person-years for males and 71.56/1,000 person-years for females).

### Characteristics of participant study

The results of the analysis are shown in Table 1 (at the end of the manuscript). The analysis revealed that the proportions of junior, senior, and secondary were the highest in both the case and control groups, at 78.5 and 71.1%, respectively. The proportion of people with a junior college or above in the case group was 20.4%, lower than 27.6% in the control group, with a statistically significant difference (*P* < 0.05). The proportions of workers in the steel enterprise with a per capita monthly income of RMB1,500—and RMB ≥ 2,500 were 46.8 and 32.6%, respectively, higher than those of the control group at 41.0% and 25.6%, respectively, with statistically significant differences (*P* < 0.001). Regarding the health status of steelworkers, the proportions of overweight, obesity, hypertension, diabetes, dyslipidemia, abnormal kidney function, and abnormal liver function in the case group were all higher than those in the control group, with statistically significant differences (*P* < 0.05 for all diseases). In terms of behavioral lifestyle, the proportion of workers in the steel company who consumed fruit daily was the highest in both the case (51.6%) and control (59.3%) groups, and was lower in the case group than in the control group, showing a statistically significant difference (*P* < 0.05). The proportion of workers in the steel company who consumed meat daily was 33.4% in the case group, higher than that in the control group at 24.2%, with a statistically significant difference (*P* < 0.001). The proportion of workers in the steel company who consumed seafood daily was 11.4% in the case group, compared with 6.4% in the control group, hence the difference was statistically significant (*P* < 0.05). The proportions of heavy physical activities and physical exercises 5–7 times per week were 75.2 and 14.8% in the case group, respectively, compared with 82.2 and 22.9% in the control group, presenting statistically significant differences (*P* < 0.001 for both). The proportion of alcohol consumption in the case group was 53.8%, higher than that of 40.1% in the control group, showing a statistically significant difference (*P* < 0.001). The differences in the distribution of marital status, frequency of vegetable consumption, frequency of egg consumption, frequency of soy product consumption, smoking, and tea-drinking between the case and control groups were not statistically significant (*P* > 0.05 for all parameters).

**Table 1:** Comparison of population characteristics of steelworkers

Variable	Control group	Case group	χ^2^	*P*
Marital status, n (%)			1.395	0.498
Unmarried	19(3.0)	13(2.0)		
Married	594(92.7)	596(93.0)		
Other	28(4.3)	32(5.0)		
Education level, n (%)			9.240	0.010*
Primary and below	8(1.3)	7(1.1)		
Junior, Senior and Secondary	456(71.1)	503(78.5)		
Junior college or above	177(27.6)	131(20.4)		
Monthly per capita household income (RMB), n (%)			27.294	< 0.001*
< 1,500	214(33.4)	132(20.6)		
1,500–2,500	263(41.0)	300(46.8)		
≥ 2,500	164(25.6)	209(32.6)		
BMI (kg/m^2^), n (%)			61.599	< 0.001*
< 24	340(53.1)	206(32.1)		
24–27	220(34.3)	286(44.6)		
≥ 28	81(12.6)	149(23.3)		
High blood pressure, n (%)			23.505	< 0.001*
No	579(90.3)	518(80.8)		
Yes	62(9.7)	123(19.2)		
Diabetes, n (%)			20.182	< 0.001*
No	591(92.2)	539(84.1)		
Yes	50(7.8)	102(15.9)		
Dyslipidemia, n (%)			29.845	< 0.001*
No	279(43.5)	185(28.9)		
Yes	362(56.5)	456(71.1)		
Abnormal kidney function, n (%)			18.293	< 0.001*
No	563(87.8)	506(78.9)		
Yes	78(12.2)	135(21.1)		
Abnormal liver function, n (%)			6.086	0.014*
No	550(85.8)	517(80.7)		
Yes	91(14.2)	124(19.3)		
Frequency of vegetable consumption, n (%)			5.255	0.072
Occasionally	46(7.2)	46(7.2)		
Frequently	229(35.7)	268(41.8)		
Daily	366(57.1)	327(51.0)		
Frequency of fruit consumption, n (%)			9.152	0.010*
Occasionally	49(7.6)	46(7.2)		
Frequently	212(33.1)	264(41.2)		
Daily	380(59.3)	331(51.6)		
Frequency of meat consumption, n (%)			18.146	< 0.001*
Never	11(1.7)	16(2.5)		
Occasionally	235(36.7)	228(35.6)		
Frequently	240(37.4)	183(28.5)		
Daily	155(24.2)	214(33.4)		
Frequency of egg consumption, n (%)			7.513	0.057
Never	76(11.9)	99(15.5)		
Occasionally	432(67.4)	441(68.8)		
Frequently	115(17.9)	88(13.7)		
Daily	18(2.8)	13(2.0)		
Frequency of dairy product consumption, n (%)			11.601	0.009*
Never	12(1.8)	19(3.0)		
Occasionally	192(30.0)	217(33.9)		
Frequently	341(53.2)	283(44.1)		
Daily	96(15.0)	122(19.0)		
Frequency of soy product consumption, n (%)			3.329	0.344
Never	28(4.3)	29(4.5)		
Occasionally	403(62.9)	423(66.0)		
Frequently	189(29.5)	162(25.3)		
Daily	21(3.3)	27(4.2)		
Frequency of seafood consumption, n (%)			17.202	0.001*
Never	106(16.5)	75 (11.7)		
Occasionally	340(53.1)	314(49.0)		
Frequently	154(24.0)	179(27.9)		
Daily	41(6.4)	73(11.4)		
Physical activity level, n (%)			20.026	< 0.001*
Light physical activity	54(8.4)	107(16.7)		
Moderate physical activity	60(9.4)	52(8.1)		
Heavy physical activity	527(82.2)	482(75.2)		
Physical exercise, n (%)			20.870	< 0.001*
Never exercise	132(20.6)	187(29.2)		
1 to 2 times a week	299(46.7)	301(47.0)		
3 to 4 times a week	63(9.8)	58(9.0)		
5 to 7 times a week	147(22.9)	95(14.8)		
Smoking, n (%)			1.394	0.498
Never smoked	274(42.8)	256(39.9)		
Former smoker	33(5.1)	30(4.7)		
Now smoking	334(52.1)	355(55.4)		
Drinking, n (%)			24.252	< 0.001*
No	384(59.9)	296(46.2)		
Yes	257(40.1)	345(53.8)		
Tea drinking, n (%)			2.195	0.138
No	270(42.1)	244(38.1)		
Yes	371(57.9)	397(61.9)		

^*^indicates statistical significance

### Single-factor analysis of the exposure to occupational hazards

A comparison of the distribution of exposure to occupational hazards between the case group and the control group revealed that ever shifts, current shifts, heat exposure, and dust exposure differed significantly between the two groups, but the differences were not statistically significant in the two noise exposure groups. Please refer to Table 2.

**Table 2:** Comparison of exposure to different occupational hazardous factors

Variable	Control group	Case group	χ^2^	*P*
Shift work, n (%)			10.129	0.006*
Never	97(15.1)	61(9.5)		
Ever	132(20.6)	154(24.0)		
Current	412(64.3)	426(66.5)		
Heat exposure, n (%)			11.252	0.001*
No	355(55.4)	277(43.2)		
Yes	286(44.6)	364(56.8)		
Dust exposure, n (%)			13.873	< 0.001*
No	457(71.3)	394(61.5)		
Yes	184(28.7)	247(38.5)		
Noise exposure, n (%)			0.383	0.536
No	288(44.9)	277(43.2)		
Yes	353(55.1)	364(56.8)		

^*^indicates statistical significance

### Univariate analysis of the cumulative exposure to occupational hazards

In the cumulative exposure, those not exposed to occupational hazards with a cumulative exposure of 0 formed a group. The median among exposures to occupational hazards was obtained. With 0 and the median as bounds, each cumulative exposure was divided into three groups (0,0-median, ≥ median). The median cumulative exposure to night shifts in the shift work population was 1,972.80. The median cumulative exposure to heat in the heat-exposed population was 567.83. The median cumulative exposure to dust in the dust-exposed population was 30.02. The median cumulative exposure to noise in the noise-exposed population was 1,660.68.

After comparing the distribution of cumulative exposure to occupational hazards between the case and control groups, we found that the proportion of the cumulative number of days of night work in the case group was 46.5% for ≥ 1,972.80 day, which was higher than that of the control group (41.2%), presenting a statistically significant difference (*P* < 0.05). The proportions of cumulative exposure to heat in the case group were 26.1% and 30.7% for 0–567.83(°C/year) and ≥ 567.83(°C/year), respectively, which were higher than those of the control group (24.6% and 20.0%), which shows statistically significant differences (*P* < 0.001). The difference in the cumulative dust exposure between the two groups was also statistically significant (*P* < 0.05). The difference in the cumulative noise exposure between the two groups was not statistically significant (*P* > 0.05). Please see Table 3.

**Table 3:** Distribution of the cumulative occupational exposure after segmentation

Variable	Control group	Case group	χ^2^	*P*
Cumulative number of days of night work (day), n (%)			10.267	0.006*
0	97(15.1)	143(9.5)		
0–1,972.80	280(43.7)	173(44.0)		
≥ 1,972.80	264(41.2)	168(46.5)		
Cumulative exposure to heat (°C/year), n (%)			24.525	< 0.001*
0	355(54.4)	277(43.2)		
0–567.83	158(24.6)	167(26.1)		
≥ 567.83	128(20.0)	197(30.7)		
Cumulative exposure to dust (mg/m^3^/year), n (%)			14.532	0.001*
0	457(71.3)	394(61.5)		
0–30.02	96(15.0)	119(18.5)		
≥ 30.02	88(13.7)	128(20.0)		
Cumulative noise exposure (dB(A)/year), n (%)			0.886	0.642
0	288(44.9)	277(43.2)		
0–1,660.68	181(28.2)	177(27.6)		
≥ 1,660.68	172(26.8)	187(29.2)		

^*^indicates statistical significance

### Multi-factor analysis of the effect of occupational hazard exposure on HUA in workers in the steel company

Table 4 shows the analysis results of the effect of occupational hazard exposure on HUA by dint of the Conditional Logistic Regression model. The dependent variable was the presence or absence of hyperuricemia, and the independent variable was the occupational exposure to harmful factors. After the adjustment of possible confounders, the OR (95% *CI*) for the risk of developing hyperuricemia was 2.18 (1.28–3.69) times and 1.81 (1.11–2.95) times for ever shifts and current shifts, respectively, compared to the reference group. Heat exposure and dust exposure were associated with an elevated risk of developing hyperuricemia with ORs (95% *CI*) of 1.58 (1.17–2.14) and 1.34(1.01–1.81), respectively. In addition, the effect of noise exposure on the risk of hyperuricemia development in steelworkers was not statistically significant (*P* > 0.05).

**Table 4:** Logistic regression analysis of the connection between the exposure to occupational hazards and HUA

Variable	Model I		Model II		Model III	
	**OR (95% *CI*)**	***P***	**OR (95% *CI*)**	***P***	**OR (95% *CI*)**	***P***
Shift work						
Never	1.00	-	1.00	-	1.00	-
Ever	2.00(1.31–3.05)	0.001*	2.01(1.25–3.22)	0.004*	2.18(1.28–3.69)	0.004*
Current	1.69(1.15–2.49)	0.008*	1.68(1.09–2.60)	0.020*	1.81(1.11–2.95)	0.018*
Heat exposure						
No	1.00	-	1.00	-	1.00	-
Yes	1.63(1.27–2.08)	< 0.001*	1.47(1.13–1.93)	0.005*	1.58(1.17–2.14)	0.003*
Dust exposure						
No	1.00	-	1.00	-	1.00	-
Yes	1.43(1.11–1.83)	0.005*	1.48(1.12–1.94)	0.005*	1.34(1.01–1.81)	0.048*
Noise exposure						
No	1.00	-	1.00	-	1.00	-
Yes	0.91(0.71–1.16)	0.446	0.93(0.71–1.23)	0.625	0.95(0.70–1.29)	0.759

^*^indicates statistical significance

Model I: Adjusted for service years, marital status, education level, and monthly per capita household income

Model II: Adjusted for BMI, hypertension, diabetes, dyslipidemia, abnormal kidney function, and abnormal liver function based on Model I

Model III: Adjusted for frequency of vegetable consumption, frequency of fruit consumption, frequency of meat consumption, frequency of egg consumption, frequency of dairy product consumption, frequency of soy product consumption, frequency of seafood consumption, physical activity level, physical exercise, smoking, drinking, tea drinking based on Model II

### Multi-factor analysis of the effect of the cumulative exposure to occupational hazards on the HUA of workers in the steel company

Cumulative exposure to occupational hazards was segmented as above. A Conditional Logistic Regression model was employed with the presence or absence of hyperuricemia as the dependent variable, and the segmented cumulative exposure to occupational hazards as the independent variable. After the adjustment of possible confounders, the results showed an increased risk of developing hyperuricemia in the cumulative days of night work in the 0–1,972.80 and ≥ 1,972.80 (days) compared to the reference group, with ORs (95% *CI*) of 1.87(1.12–3.13) and 2.02(1.21–3.37), respectively. The ORs (95% *CI*) for the risk of developing hyperuricemia in steelworkers in the cumulative exposure to heat groups of 0–567.83 and ≥ 567.83 (°C/year) were 1.50(1.02–2.22) and 1.64(1.11–2.43), respectively. Compared to the reference group, the OR (95% *CI*) for the risk of hyperuricemia in steelworkers was 1.56 (1.05–2.32) for cumulative dust exposure ≥ 30.02 (mg/m^3^/year). The OR (95% *CI*) for the risk of developing hyperuricemia in steelworkers at dust exposure of ≥ 30.02 (mg/m^3^/year) was 1.16(0.78–1.73), which was not statistically significant (*P* > 0.05) compared to the reference group. The association between cumulative noise exposure and hyperuricemia development in steelworkers was not statistically significant (*P* > 0.05). Please refer to Table 5.

**Table 5:** Logistic regression analysis of the connection between post-occupational cumulative exposure segmentation and HUA

Variable	Model I		Model II		Model III	
	**OR (95% *CI*)**	***P***	**OR (95% *CI*)**	***P***	**OR (95% *CI*)**	***P***
Cumulative number of days of night work (day)						
0	1.00	-	1.00	-	1.00	-
0–1,972.80	1.76(1.16–2.66)	0.007*	1.71(1.08–2.71)	0.023*	1.87(1.12–3.13)	0.017*
≥ 1,972.80	1.87(1.25–2.80)	0.002*	1.92(1.22–3.01)	0.005*	2.02(1.21–3.37)	0.007*
Cumulative exposure to heat (°C/year)						
0	1.00	-	1.00	-	1.00	-
0–567.83	1.31(0.95–1.80)	0.095	1.42(1.00–2.01)	0.051	1.50(1.02–2.22)	0.041*
≥ 567.83	1.95(1.43–2.66)	< 0.001*	1.53(1.08–2.17)	0.016*	1.64(1.11–2.43)	0.014*
Cumulative exposure to dust (mg/m^3^/year)						
0	1.00	-	1.00	-	1.00	-
0–30.02	1.23(0.89–1.70)	0.214	1.27(0.89–1.80)	0.192	1.16(0.78–1.73)	0.459
≥ 30.02	1.69(1.21–2.36)	0.002*	1.75(1.21–2.52)	0.003*	1.56(1.05–2.32)	0.028*
Cumulative noise exposure (dB(A)/year)						
0	1.00	-	1.00	-	1.00	-
0–1,660.68	0.91(0.65–1.26)	0.563	0.87(0.60–1.26)	0.462	0.91(0.61–1.37)	0.653
≥ 1,660.68	0.94(0.70–1.27)	0.696	0.97(0.70–1.34)	0.845	0.97(0.68–1.38)	0.851

^*^indicates statistical significance

Model I: Adjusted for service years, marital status, education level, and monthly per capita household income

Model II: Adjusted for BMI, hypertension, diabetes, dyslipidemia, abnormal kidney function, and abnormal liver function based on Model I

Model III: Adjusted for frequency of vegetable consumption, frequency of fruit consumption, frequency of meat consumption, frequency of egg consumption, frequency of dairy product consumption, frequency of soy product consumption, frequency of seafood consumption, physical activity level, physical exercise, smoking, drinking, tea drinking based on Model II

### Correlation of interactions between occupational hazards and HUA

The multiplicative interactions between shift work, heat exposure, and dust exposure were analyzed in the case of hyperuricemia. The main effects of the two occupational hazards and the product terms of the two multiplicative interactions were jointly introduced into the Conditional Logistic Regression model for analysis. The model analysis was adjusted for service years, marital status, education level, monthly per capita household income, physical activity level, BMI, hypertension, diabetes, dyslipidemia, abnormal kidney function, abnormal liver function, frequency of vegetable consumption, frequency of fruit consumption, frequency of egg consumption, frequency of dairy consumption, frequency of soya product consumption, frequency of seafood consumption, physical exercise, smoking, drinking, and tea drinking. The results showed a multiplicative interaction between dust exposure and heat exposure (*P *_*interaction*_ < 0.05). Dust exposure significantly increased the effect of heat exposure on hyperuricemia development (OR = 2.45, 95% *CI*: 1.34–4.46). No multiplicative interactions were found between shift work and heat exposure or shift work and dust exposure (*P *_*interactions*_ > 0.05 for all variables). Please see Tables 6, 7 and 8.

**Table 6:** Analysis of the multiplicative interaction between shift work and heat exposure on HUA

Variable	OR (95% *CI*)	*P*
Never shifts	1.00	-
Ever shifts	3.30(1.61–6.79)	0.001
Current shifts	2.40(1.25–4.61)	0.008
Heat exposure	3.04(1.21–7.61)	0.018
Never shifts × heat exposure	1.00	-
Ever shifts × heat exposure	0.41(0.13–1.25)	0.409
Current shifts × heat exposure	0.53(0.20–1.42)	0.533

**Table 7:** Analysis of the multiplicative interaction between shift work and dust exposure on HUA

Variable	OR (95% *CI*)	*P*
Never shifts	1.00	-
Ever shifts	2.49(1.33–4.66)	0.004
Current shifts	1.88(1.07–3.29)	0.029
Dust exposure	1.69(0.65–4.40)	0.280
Never shifts × dust exposure	1.00	-
Ever shifts × dust exposure	0.61(0.20–1.88)	0.387
Current shifts × dust exposure	0.91(0.33–2.47)	0.846

**Table 8:** Analysis of the multiplicative interaction between heat exposure and dust exposure on HUA

Variable	OR (95% *CI*)	*P*
Heat exposure	1.17(0.82–1.67)	0.382
Dust exposure	0.85(0.55–1.33)	0.485
Heat exposure × dust exposure	2.45(1.34–4.46)	0.003*

^*^indicates statistical significance

Since the additive interaction model can only be employed to explore interactions between dichotomous variables, the ever shifts and current shifts were combined into one group. The shift states were divided into never-shift and shift for the analysis of additive interactions. The results revealed no additive interactions between shift work and heat exposure, shift work and dust exposure, or heat exposure and dust exposure. Please refer to Table 9.

**Table 9:** Analysis of additive interactions between occupational hazards on HUA in steelworkers

Variable	RERI (95% *CI*)	AP (95% *CI*)	SI (95% *CI*)
Shift work^a^ × heat exposure	-0.170(-0.192–1.152)	-0.061(-0.533–0.410)	0.912(0.467–1.783)
Shift work^a^ × dust exposure	-0.464(-2.122–1.194)	-0.170(-0.773–0.432)	0.788(0.372–1.670)
Heat exposure × dust exposure	1.464(0.677–2.251)	0.558 (0.362–0.754)	10.250(0.394–266.490)

^a^Dichotomous variables were used for the analysis. Ever shifts and current shifts were combined as shifts. The shift work was divided into never-shifts and shifts

## Discussion

With rapid socio-economic development and changes in people’s lifestyles, hyperuricemia has become the second most prevalent metabolic disease after diabetes, seriously affecting people’s quality of life [29]. Hyperuricemia is a risk factor for many diseases. The deposition of urate crystals can give rise to metabolic disorders and damage to kidney and heart [30, 31]. Studies have shown that the prevalence of hyperuricemia in adults is increasing year by year. There is a trend of a younger onset of the disease [9].

In this study, the incidence rate of hyperuricemia in the workers of the steel company investigated was 17.30%. A meta-analysis revealed that 13.3% of people in the mainland of China had hyperuricemia [9]. The incidence rate of hyperuricemia among workers in steel companies is higher than the average in the mainland of China. It is therefore essential to study the effects of occupational exposure on hyperuricemia in steelworkers.

According to the US 2010 National Health Interview Survey, about one-fifth of the workforce carries out shift work of varying intensities worldwide [32]. Shift work has many adverse effects on the physical and mental health of workers. Therefore, shift work is one of the occupational hazards that cannot be overlooked. Despite production reforms, shift work is still practiced in the steel industry. Of the 641 sample pairs in this study, 87.7% of workers in the steel enterprise had a history of shift work, and 65.4% of workers in the steel enterprise were currently working in shifts. In this study, after the adjustment of possible confounders, it was found that the ORs (95% CI) for the risk of developing hyperuricemia due to ever shifts and current shifts were 2.18 and 1.81 times higher than that due to never shifts, respectively. The risk of hyperuricemia was increased in both the 0–1,972.80 and ≥ 1,972.80 (days) groups compared to the 0 (day) cumulative days of night work. The ORs were 1.87 and 2.02, respectively, indicating that the risk of developing hyperuricemia rose with the number of cumulative days of night work. A Japanese cohort study revealed that shift work is independently related to elevated serum uric acid in males [33]. In another study, the risk of hyperuricemia development was 1.41 times higher in steelworkers who worked shifts compared to that of those who didn’t [34]. The present study echoes with these results. Long-term shift work disrupts physiological functions and the body’s circadian rhythm. At the same time, the biological clock is disturbed, thus impairing uric acid metabolism. Studies suggest that the culprit could be oxidative stress caused by disrupted circadian rhythms [35].

Heat is among the main occupational hazards for workers in steel companies, and high workplace temperatures can place a burden of disease on occupational groups [36, 37]. Over 50% of the 641 sample pairs in this study were exposed to heat. The current study showed that heat exposure raised the risk of hyperuricemia development with an OR of 1.58. The risk of developing hyperuricemia was increased in both groups with the cumulative exposure to heat of 0–567.83 and ≥ 567.83 (°C/year) compared to the reference group, respectively. The ORs were 1.50 and 1.64, respectively. Lin QY et al. examined the factors impacting the prevalence of chronic diseases among heat-exposed workers in a port terminal, and the results revealed that the longer the length of labor exposed to heat, the higher the risk of hyperuricemia is (*P* < 0.01), which is similar to the results of this study [38]. The mechanisms involved are hypothesized: firstly, under the hot working conditions, most of the water in the body is excreted in sweat, thus the urinary excretion is significantly reduced and uric acid accumulates. Secondly, the concentration of lactic acid in workers’ bodies rises under high-temperature working conditions. Lactic acid competitively inhibits the excretion of uric acid. The competitive inhibition affects uric acid excretion and the concentration of uric acid increases in the blood. Furthermore, research has demonstrated that exposure to heat might lead to kidney damage [11]. Kidney damage can affect the normal excretion of uric acid, leading to the accumulation of uric acid and, ultimately, hyperuricemia.

Dust is generated in all links of the steel production process, and exposure to dust is one of the major occupational hazards for steelworkers. This study showed that dust exposure elevated the risk of developing hyperuricemia in comparison to no exposure to dust with an OR of 1.34. The risk of hyperuricemia development was increased in the group with cumulative dust exposure ≥ 30.02 (mg/m^3^/year) compared to the reference group. The OR (95% *CI*) was 1.56 (1.05–2.32). When dust is inhaled by the body, it not only accumulates in the lungs, but also enters the circulation through the blood barrier and damages other organs [39]. This has been confirmed by many animal tests [40, 41]. It is, therefore, speculated that the increased risk of hyperuricemia development from dust exposure may be owing to kidney damage, which affects the normal metabolism of purines and the normal excretion of uric acid, leading to an elevation in uric acid levels in the blood. There are fewer studies on the association between dust and hyperuricemia, and more research is needed to make clarify the exact mechanisms.

The present study showed no statistically significant association between noise exposure and hyperuricemia, which is consistent with the findings of the Zhang SK study [42]. However, it has also been noted that hyperuricemia is associated with noise exposure in the work environment [43]. Noise-induced psychological stress may affect purine metabolism and uric acid excretion through neuroendocrine regulation [44]. Therefore, the effect of noise exposure on hyperuricemia requires additional investigation.

Few research has clarified the interplay of occupational hazards in prior studies on factors affecting hyperuricemia. In this study, the interaction analysis revealed a multiplicative interaction between heat exposure and dust exposure in the development of hyperuricemia. Exposure to both heat and dust significantly increased the risk of hyperuricemia development, which proves the combined effect of some occupational hazards on physical health. This indicates that reducing workers’ heat exposure can lower the risk of hyperuricemia in steelworkers exposed to dust so as to protect their health.

The main strengths of our study lie in a precise calculation of cumulative exposure to occupational hazards and a comprehensive range of potential confounders, which enables a more accurate study on the effect of occupational exposures on hyperuricemia. However, there are some limitations in our study. There was a 12.74% missed visit rate throughout the study, which may have been subject to missed visit bias. High-temperature and dusty weathers may affect the results of this study, but they weren’t taken into account in this study. Moreover, this study is only based on a sample of workers from a steel company in one region. Due to the uniqueness of the occupational environment, the sample size of female workers was small and the representation of the study population was limited. Therefore, a multi-regional and large sample of the target population is required for validation.

## Conclusion

Shift work, heat, and dust are three independent factors that put steelworkers at risk for hyperuricemia, and exposure to both heat and dust increases the risk of hyperuricemia development. Therefore, close monitoring of the aforementioned factors and early intervention are required to lower the prevalence of hyperuricemia and improve the health of steelworkers.

## Data Availability

Data are available upon reasonable request. The datasets generated and analyzed during the current study are not publicly available due other analyses are proceeding but are available from the corresponding author on reasonable request.

## Abbreviations

HUA: Hyperuricemia
BMI: Body Mass Index
OR: Odds ratio
95%*CI*: 95% Confident limit
RERI: Relative excess risk of interaction
AP: Attributable proportion of interaction
SI: Synergy index
